# Formulation Optimization of Sustained-Release Ammonio Methacrylate Copolymer Microspheres. Effects of Log P and Concentration of Polar Cosolvents, and Role of the Drug/Copolymer Ratio

**DOI:** 10.3390/pharmaceutics3040830

**Published:** 2011-11-10

**Authors:** Péter Sipos, Róbert Rajkó, Klára Pintye-Hódi, István Erős, Piroska Szabó-Révész

**Affiliations:** 1 Department of Pharmaceutical Technology, University of Szeged, Eotvos str. 6, 6720 Szeged, Hungary; 2 Department of Mechanical and Process Engineering, University of Szeged, Dugonics squ. 13, 6720 Szeged, Hungary

**Keywords:** microspheres, formulation optimization, cosolvents, drug/copolymer ratio, spray drying, factorial design study

## Abstract

The objectives of this work were the formulation optimization of the preparation process parameters and to evaluate spray-dried sustained-release microspheres using ammonio methacrylate copolymer (AMC) as a polymer matrix. The effects of log P and the concentrations of the cosolvents (acetone, methyl ethyl ketone and *n*-butyl acetate) and different drug/copolymer ratios as independent variables on the physicochemical parameters (the W_1_/O emulsion viscosity, the microsphere production yield, the average particle size, the encapsulation efficiency) and the cumulative *in vitro* drug release as dependent variables were studied. The optimization was carried out on the basis of the 3^3^ factorial design study. The optimization process results showed that addition of polar cosolvents proved effective, linear relationships were observed between the independent and the dependent variables. The best conditions were achieved by microspheres prepared by using a low/medium cosolvent log P, cosolvent concentration of 25–50% v/v and a drug/copolymer ratio of 1:16. The microspheres ensured sustained release with Nernst and Baker-Lonsdale release profiles.

## 1. Introduction

The value of microparticulate delivery systems as orally administered controlled-release dosage forms has been evident for years. Microencapsulation is used for extending the drug action, to control drug release kinetics, minimize side effects and reduce gastric irritations. Appropriate preparation techniques should be designed and complex investigations of the effects of the emphasized physicochemical factors should be performed to overcome the drawbacks of the microparticles. Optimization of the preparation process is advantageous for efficient drug entrapment; the factors may alter the distribution of the microparticle parameters markedly, determining the drug release mechanism.

Selection of the organic solvent may determine drug characteristics such as crystal form and solubility. The addition of a polar cosolvent to CH_2_Cl_2_ or replacement of CH_2_Cl_2_ may act in two different ways: increasing the polymer precipitation rate and at the same time decreasing the encapsulation efficiency (EE), due to the confluence of the aqueous phases of the multiple emulsion; thus, there can be a sensitive balance between these opposite effects. The integrity of the forming microsphere wall is controlled by the rate of migration of the organic solvent to the outer aqueous phase and also by the rate of evaporation from this phase. The rate of solvent extraction is limited by the water-solubility of the organic solvent used, while the evaporation rate depends on its boiling point. At the water-organic phase interface, cosolvents with low affinity for the polymer are the first to diffuse out from the W_1_/O emulsion droplet, depending on their physicochemical properties [1]. The use of organic solvents in order to prepare microspheres has been investigated previously, Class 2 solvents according to ICH e.g., chloroform [2], 1,2-dichloroethane [3], cyclohexane [4], dichloromethane (CH_2_Cl_2_) [5,6] and methanol [7,8]; and Class 3 solvents as acetone (Me_2_CO) [8,9], ethanol [10,11], and ethyl acetate [12]. Removal of organic solvents during manufacturing is critical, and it is therefore an industrial requirement to test the amount of residual organic solvents.

The model drug (diclofenac sodium) was a non-steroidal anti-inflammatory drug. In consequence of its short half-life and therefore the need for multiple dosing, which involves the increased risk of adverse effects, the achievement of sustained release is of great importance. Diclofenac sodium carriers have been investigated to improve the pharmacological efficiency, as alginate microspheres [13], thermo-responsive gelatine microspheres [14], compressed matrix pellets [15], alginate/hydroxyapatite composite beads [16] and liposomes [17]. The polymer component applied was biocompatible and non-biodegradable ammonio methacrylate copolymer (AMC), which has been used as a retardant in the formulation of sustained-release pellets [18], matrix tablets [19], thermosensitive membranes [20], microparticles [21], tablet coatings [22], chitosan particle coating [11] and spray-dried vaccine carriers [23]. The main advantages expected from AMC are insolubility in the digestive juice and a pH-independent slow release, which is diffusion-controlled through the polymer wall and the pores formed during the droplet-hardening process [24].

The protective colloid was poly(vinyl alcohol) to prevent the W_1_/O droplets from coalescing, and the amphiphilic polyethylene glycol stearate was applied as a plasticizer of the copolymer in order to make the polymer chains more flexible [25]. To improve the stability of the W_1_/O/W_2_ emulsions, nonionic surfactants were used. One of the methods of preparation of microparticles is the spray-drying of the drug-polymer solution [26] or multiple emulsion [27,28]. During the spray-drying process, the rapid solvent evaporation leads to quick polymer solidification, resulting in a higher EE, and it can be used to minimize the time necessary for microsphere formation.

The main objective was to optimize the formulation parameters of drug containing matrix system with a sustained release profile, to ensure microsphere product quality and a potential for scaling-up for further investigations towards oral administration. The present work was designed to evaluate the effects of three Class 3 cosolvents on the characteristics of the microspheres. A 3^3^ factorial design was used to investigate the effects of three independent variables: log P, the concentrations of Class 3 polar cosolvents, and different drug/copolymer ratios.

Although the release profile is a useful feedback for the evaluation and recognition of coherences in matrix systems, it is complicated to draw conclusions regarding the structure of the microspheres from the release profiles without an adequate amount of supporting evidence [29]. Accordingly, the external morphology, the production yield, average particle size, encapsulation efficiency (EE), and the cumulative percentage drug release (Q_6_) were measured as dependent variables. The required parameters were low W_1_/O emulsion viscosity (η) and particle size; relatively high production yield and EE; and Q_6_ value in the ranges 40–80% in 6 h.

## 2. Experimental Section

### 2.1. Materials

Diclofenac sodium (Ph.Eur. 7) was used as hydrophilic model drug (Sigma, Hungary). Ammonio Methacrylate Copolymer Type B (AMC, MW 150 000) (Ph.Eur. 7) was used as matrix-forming copolymer (Eudragit; Evonik Ind., Essen, Germany). The solvent dichloromethane (CH_2_Cl_2_) and the cosolvents, acetone (Me_2_CO), methyl ethyl ketone (MeCOEt) and *n*-butyl acetate (*n*BuOAc) were of reagent grade (Sigma, Hungary). At the concentrations used, the cosolvents are water-miscible (Me_2_CO) or partially miscible (MeCOEt and *n*BuOAc), their physicochemical data were from chemical databases (Table 1). The non-ionic surfactants sorbitan mono-oleate and polyoxyethylene 20 sorbitan mono-oleate (MW = 428 and 1309, respectively), the protective carrier poly(vinyl alcohol) (MW = 72 000) and the plasticizer PEG stearate were of pharmacopoeial grade (MW = 328) (Ph.Eur. 7) (VWR Co., Hungary).

### 2.2. Preparation of Microspheres

A W_1_/O/W_2_ multiple emulsion was prepared by a two-step emulsification procedure. An aqueous solution of the drug was dispersed by homogenization (17 600 rpm, 5 min) (Heidolph Diax 900, Heidolph, Germany) into the organic phase, which consisted of CH_2_Cl_2_ or a CH_2_Cl_2_-cosolvent mixture, the copolymer, plasticizer and emulsifier. The resulting W_1_/O emulsion was added to the W_2_ phase, containing protective carrier and the O/W emulsifier, under continuous stirring at 1500 rpm. The multiple emulsion was then spray-dried using a Büchi B-191 Laboratory Spray-dryer (Büchi Co., Flawil, Switzerland) with a standard 0.7 mm nozzle. The microspheres were separated in the novel high-performance cyclone. Spray-drying was carried out under the same conditions: at 11.6 1 min^−1^ air flow, 5 bar pressure, and 2.1 mL·min^−1^ pump flow rate. The inlet temperature was set above the boiling points of the cosolvents (125 °C), and the outlet temperature varied in the range 89 ± 5 °C. The final products were stored under controlled humidity conditions at 4 °C. The preparation yield was calculated as the ratio of the actual and theoretical masses of the microsphere batch.

### 2.3. W_1_/O Primary Emulsion Characterization

The dynamic viscosity of the W_1_/O emulsions (η) were measured at 20 ± 1 °C with a rotational viscometer (Brookfield DV-III, Brookfield Co., UK), at a constant shear rate of 130 1 s^−1^ (n = 5). Immediately after preparation of all the examined W_1_/O/W_2_ emulsions, without dilution, microscopic observations were made with a LEICA LaborLux S image analyser at 100× magnification (LEICA Co., Germany).

### 2.4. Microsphere Characterization

#### 2.4.1. Particle Size Analysis

Microspheres (0.5 g) were suspended in surfactant-free deionized water, and sized by laser diffractometry without sonication, using a Malvern Mastersizer laser sizer (Malvern Instruments, Malvern, UK) (n = 5). The weighted average of the volume distribution (*D [4.3]*) was used to describe particle size (μm). The width of the particle size distributions was expressed by the SPAN parameter (width of the particle size distribution based on the 10%, 50% and 90% quantile).

#### 2.4.2. Morphological Study by Scanning Electron Microscopy

Surface characteristics and external morphology were determined by SEM, using the Hitachi S2400 instrument (Hitachi Scientific Instruments, Tokyo, Japan). A Polaron sputter coating apparatus (Polaron Equipment, Greenhill, UK) was applied to induce electric conductivity on the surface of the sample. The air pressure was 1.3–13 mPa. The surface and shape structure were examined with a Hitachi S2400 instrument (Hitachi Scientific Instruments, Tokyo, Japan).

#### 2.4.3. Determination of Drug Encapsulation Efficiency

The encapsulation efficiency (EE, %) was determined with an energy-dispersive X-ray fluorescence analyser (MiniPal, Philips Analytical, The Netherlands). The spectrum was evaluated by non-linear least squares fitting. Pressed microsphere samples were prepared (n = 7). X-ray tube type: low-powered with side window; anode material: Rh; software-controlled tube setting; tube filters: 5 filters selected by software. Measurement conditions: He gas inlet pressure: 1 bar, measuring time: 600 s; conditions set: 4 kV, 1000 μA. The calibration revealed a linear model (R^2^ = 0.994, n = 9). EE was calculated as the ratio of the actual and the theoretical loading of drug as in other studies [8].

#### 2.4.4. *In Vitro* Release Study

A modified paddle Apparatus II (Ph.Eur. 7) was used for the experiments. Dissolution was performed in surfactant-free phosphate-buffered saline (PBS) (pH 7.42) at 37 ± 0.5 °C, at a mixing rate of 100 1 min^−1^. Samples were taken at given intervals and then replaced with fresh PBS. The drug content was analyzed by HPLC (HPLC system 1280, Jasco Co., Japan) method at 276 nm, after filtration with a 0.45 μm Millex PVDF filter. The analysis was performed at 25°C with a Luna RP-C18 column (USP L1, 5 μm particle diameter, 4.6 × 150 mm) (Phenomenex Inc., USA). The mobile phase consisting of methanol-NaH_2_PO_4_ buffer at pH 2.5 with ortho-phosphoric acid (66:34), was pumped at 1.0 mL min^−1^. Calibrations were made by the external standard method (R^2^ = 0.997, n = 9). Six types of kinetic models were applied to process: the zero-order and first-order release equations [30], the Higuchi square root of time equation, the Hixson-Crowell cube root model, the Baker-Lonsdale model and the Nernst equation.

#### 2.4.5. Analysis of Residual Organic Solvent

The levels of residual organic solvents and cosolvents were determined by GC analysis, using a HP 6890 GC static head-space instrument with a set of standard organic solvent concentrations. The temperatures and GC conditions were as follows for CH_2_Cl_2_, Me_2_CO and MeCOEt: injector temp. 250 °C, inlet gas: He (0.285 bar), oven initial temp.: 35 °C, oven final temp.: 240 °C, make-up gas: N_2_; detector: FID, temp.: 320 °C. The capillary column was a DB-624. The conditions for *n*BuOAc were: injector temp: 200 °C, inlet gas: He (0.285 bar), oven initial temp.: 50 °C, oven final temp.: 200 °C, make-up gas: N_2;_ detector: FID, temp.: 250 °C. The capillary column was a Stabilwax. The calibrations revealed a linear model (R^2^ ≥ 0.995, n = 9).

### 2.5. Statistical Analysis

To evaluate the contribution of each factor with different levels on responses, a 3-factor, 3-level factorial based design was conducted, using Statistica for Windows^®^ software Version 7.1 (StatSoft Inc., USA). The factors selected as independent variables were: log P of cosolvents (X_1_), cosolvent concentrations (% v/v) (X_2_) and the drug/copolymer ratios (X_3_).

The batches were distinguished by the log P value of the cosolvents. Thus, Me_2_CO (batch *S1*-*S9*), MeCOEt (batch *S10*-*S18*), or *n*BuOAc (batch *S19*-*S27*) were mixed individually with CH_2_Cl_2_. Several parameters were examined as dependent variables: η (Y_1_), the production yield (Y_2_), particle size (Y_3_), EE (Y_4_) and Q_6_ (Y_5_). Table 2 shows the levels and actual values of the independent variables. The results were confirmed and specified by analysis of variance (one-way ANOVA) (*p* < 0.05). For evaluation of the results, the correlation coefficients (R^2^) and the *p* values were calculated.

## 3. Results and discussion

### 3.1. Characterization of W1/O Emulsion Droplets

**Figure 1.**
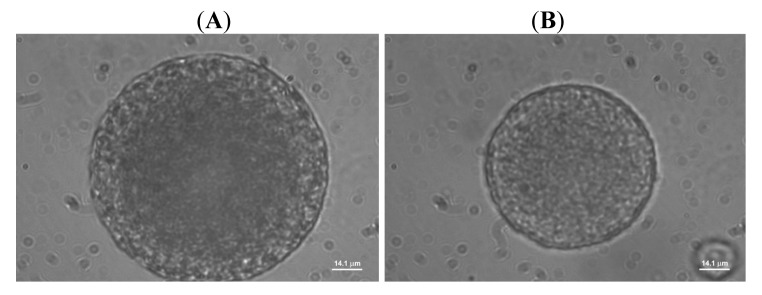

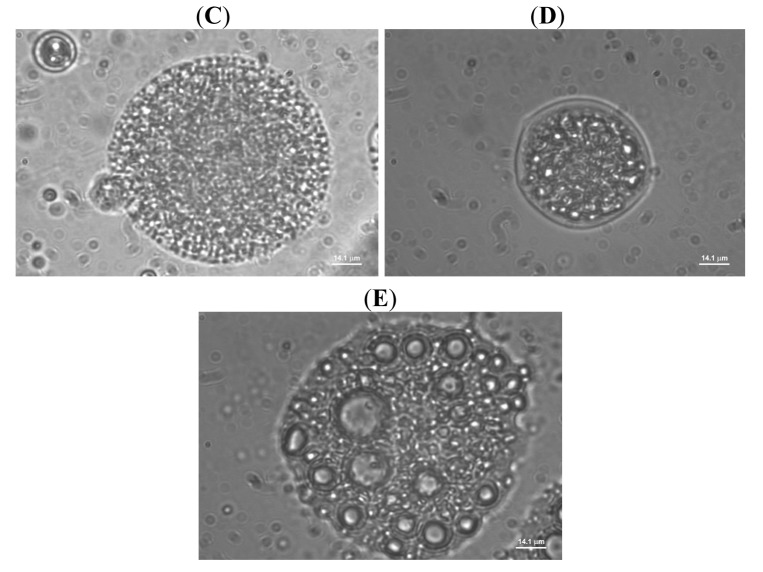
Representative image analysis of multiple emulsion droplets (magnification: 100×) (X_1_; X_2_; X_3_): (**A**) *S0A* (CH_2_Cl_2_ alone; X_3_ = −1); (**B**) *S4* (−1; 0; −1); (**C**) *S5* (−1; 0; 0); (**D**) *S6* (−1; 0; +1); (**E**) *S9* (−1; +1; +1).

The state of the W_1_/O emulsion droplets determines the morphology of the final microparticles. The W_1_/O emulsion droplet structure was changed dramatically by increasing the drug/copolymer ratio; the changes due to osmotic swelling are presented in Figure 1A–E. Increase of the drug/copolymer ratio (X_3_: −1 → +1) at a fixed volume of the cosolvent (X_2_: 0) resulted in an increase in the W_1_ droplet size due to the influx of water and merging. The emulsion droplets exhibited rupture of the interfacial layers; the physical stability therefore became critical. This alteration in the W_1_ droplet structure drastically decreased the EE value of the microspheres (*S4*-*S6*, Figure 1B–D), in accordance with the literature [31]. When the drug/copolymer ratio was fixed at 1:16 (X_3_: +1), increase of the cosolvent concentration (X_2_: 0 → +1) resulted in an increased W_1_ droplet size (*S9*, Figure 1E.). Despite of the large W_1_ droplet size, the copolymer precipitation rate increased due to the higher amount of cosolvent, increasing the EE. The trends observed for all the cosolvents used were similar.

### 3.2. SEM Evaluation of the Microspheres

**Figure 2.**
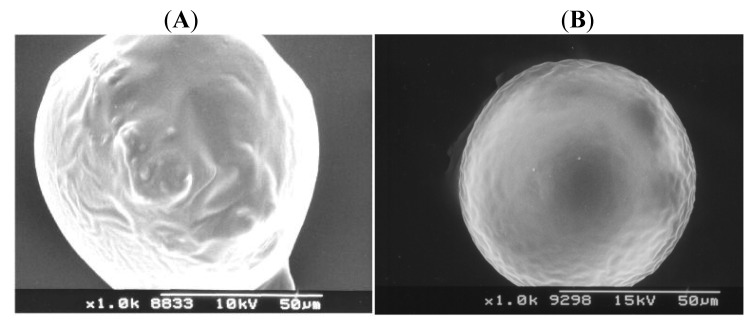

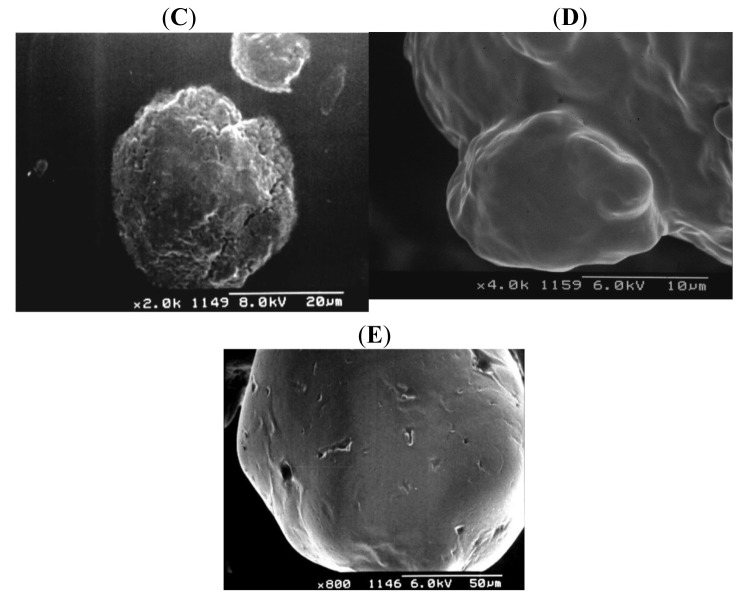
SEM evaluation of microsphere products (X_1_; X_2_; X_3_): (**A**) Drug-free sample without number (CH_2_Cl_2_ 100% v/v); (**B**) *S0C* (CH_2_Cl_2_ 100% v/v; X_3_ = +1); (**C**) *S9* (−1; +1; +1); (**D**) *S18* (0; +1; +1) and (**E**) *S27* (+1; +1; +1).

Surface characteristics and external morphology were analyzed using SEM (Figure 2A–F). The surface of the microparticle was affected by the independent variables. The rate of the emulsion droplets hardening and therefore the rate of AMC matrix preparation were different before the spray-drying. The drug-free microspheres, prepared with CH_2_Cl_2_ alone, exhibited an intact and smooth surface (Figure 2A). The drug-containing microspheres prepared with CH_2_Cl_2_ alone displayed spherical particles with a smooth surface, without agglomeration (Figure 2B).

When CH_2_Cl_2_-cosolvent mixtures were used (*S1*-*S27*), the morphology of the microspheres varied with the nature of the cosolvent. Figure 2C–E show the most critical cases when microspheres were prepared at high cosolvent concentration (X_2_: +1) and at a high drug/copolymer ratio (X_3_: +1). The trends observed for all the cosolvents used were similar. As compared the microspheres prepared with less water-soluble cosolvents (MeCOEt and *n*BuOAc), the use of Me_2_CO (Batch *S1*-*S9*) led to a dense microsphere structure, in which, despite the pores and the depressed surface, the drug release could ensure a sustained profile. As a result of rapid solvent diffusion, and therefore the fast precipitation of the copolymer, the particles were regularly shaped, but minor or gross distortions could also be observed (*S9*, Figure 2C). When MeCOEt, as a less water-soluble cosolvent, was added to CH_2_Cl_2_ (Batch *S10*-*S18*), more spherical particles with distorted surface morphology were observed, and there were several aggregated microparticles (*S18*, Figure 2D). In fact, the formation of these ‘groups of particles’ arose from the fusion of the semifinished microparticle walls at the interface, because the emulsion droplets could not be divided during the spray-drying process. The cosolvent *n*BuOAc, as the least water-soluble cosolvent, decreased te rate of solvent extraction, leading to microparticles with porous and rough surface (*S27*, Figure 2E).

### 3.3. Microspheres Prepared with CH_2_Cl_2_ 100% v/v

With increasing level of X_3_ (−1 → +1), the η decreased by 40%, from 30.9 to 18.6 mPas, particle size increased from 54 μm to 130 μm, and EE decreased considerably by 73%. The Nernst dissolution profile best followed the release profile of batch *S0A*-*S0C* (R^2^ > 0.955), and the release rate reached a plateau, after a slow dissolution. The absence of a burst effect could be due to the preferential location of the drug inside the deep sections of the copolymer matrix.

### 3.4. Y_1_ Response: Investigation of the W_1_/O Emulsion Viscosity

Table 4 shows the factorial design layout for the variables and the measured values of the responses. The precipitation of the polymer, and hence the microsphere formation, depends on the diffusion-controlled solvent removal process, the organic phase viscosity and the cosolvent concentration [32]. The viscosity is of great importance: dispersing of the droplet in the watery phase or spray-drying depends on the droplet viscosity. Batch *S19*-*S27* with the highest η ensured microspheres with a porous surface, with a low production yield, low EE value, but higher particle size and Q_6_ value with the levels of X_3_ = 0 and +1. It was observed that log P of the cosolvent (X_1_) was more of a controlling factor in the viscosity of the examined phases; however, the cosolvent concentration (X_2_) and the drug/copolymer ratio (X_3_) had significant complementary effects.

The rate of extraction of the polar cosolvent from the W_1_/O emulsion to the W_2_ phase is higher than that for CH_2_Cl_2_; thus, the organic phase viscosity increased rapidly and polymer precipitation therefore occurrs earlier. The increase of the X_2_ factor level resulted in a decreased organic phase viscosity, and therefore an increased mixing efficiency. This tendency also held true for X_3_, keeping X_2_ constant. The W_1_/O emulsion, prepared purely with CH_2_Cl_2_ (*S0A*-*S0C*) had higher η (18.6–30.9 mPas) than those of the emulsions prepared with the cosolvents examined (6.4–22.7 mPas). The lipophilic components dissolved in the CH_2_Cl_2_ + MeCOEt mixtures led to a stronger viscosity dependence than when pure CH_2_Cl_2_ and MeCOEt were mixed. At constant X_2_, η decreased with increasing X_3_, similarly in the case of Me_2_CO and *n*BuOAc. The η decreased to a larger extent at constant X_3_ with increasing X_2_; this change was statistically significant (R^2^ = 0.976, *p* = 0.002). Both the linear (b_1_) and the quadratic (b_11_) effects of the independent variables on η were statistically significant (R^2^ = 0.998, *p* < 0.008) (Table 4). X_1_ had the main (positive) effect on η (b_1_: 4.68), but the increased levels of X_2_ and X_3_ decreased it, and a synergistic interaction between X_2_ and X_3_ (b_23_: 0.53) was also observed. The required effect is a low η, which could be ensured by low and medium (−1;0) X_1_, all level of X_2_ and low level (−1) of X_3_.

### 3.5. Y_2_ Response: Investigation of Microsphere Production Yield

The production yield ranged from 26.1 to 74.6%, depending notably on the process parameters, and the viscosity and stability of the multiple emulsion to be dried. As expected, the production yield was dependent of the drug/copolymer ratio, as demonstrated by the variation between microspheres prepared at the same level of the X_3_ factor. The decrease of η led to a decrease in the efficacy of the spray-drying process and consequently in the production yield. The production yield decreased in parallel with the increase of X_1_ and X_3_. Low and medium (−1 and 0) levels of X_1_, high (+1) level of X_2_ and low level (−1) of X_3_ resulted in a higher production yield (65–72%). X_3_ was confirmed as the limiting factor, the linear (b_3_) effect of X_3_ had great influence (−6.63) (R^2^ = 0.944). It was observed that the use of *n*BuOAc and the high (+1) level of X_3_ affected the production yield most adversely. Increase of X_3_ caused a decrease in the production yield, due to the low precipitation rate of the hardening W_1_/O emulsion droplet. Low (−1) level of X_3_ demonstrated the highest production yield (45.1–62.1 %), indicating that this ratio could be used successfully at high cosolvent concentration (75% v/v) to achieve the convenient production yield during the spray-drying.

### 3.6. Y_3_ Response: Investigation of Particle Size

The particle size data were in the range of 120.6–313.4 μm (fine to moderately fine, Ph.Eur. 7.). The SPAN parameter overall varied from 1.04E+00 to 4.84E+00, reflecting a homogeneous size distribution. The rates of extraction and evaporation rate of the organic solvent and polar cosolvents are determined by their water-solubilities and boiling points, respectively. Generally, a high solvent extraction rate can lead to fast microsphere formation, no merging of the emulsion droplets and therefore a low particle size [33].

The average size (D [4,3]) of the microspheres showed a trend of decreased size with increased W_1_/O viscosity in the batches. X_1_ had high effect on particle size (b_1_ = 26.71); its increase afforded the same sequence as for the boiling points (Me_2_CO < MeCOEt ≪ *n*BuOAc) and resulted in an increased particle size, while their water-solubilities exhibited the opposite sequence. *n*BuOAc has the highest viscosity, resulting in a more viscous W_1_/O emulsion, which made it difficult to form small multiple emulsion droplets, as reported earlier [28]. *S19*-*S21* had the highest η (17.6–22.7 mPas), but did not ensure the highest particle size. In fact, *S0A* and *S0B*, prepared with CH_2_Cl_2_ alone, had relatively high η (20.5 and 30.9 mPas), with the lowest particle size (54 and 107 μm), indicating the joint effect of the independent variables. The effects of all the factors, and the quadratic effect of X_2_ (b_22_: 27.31) were found to be significant.

There was a tendency for increasing amount of drug in the W_1_ phase to lead to a decreased production yield and an increased particle size, which proved to be opposite effects. The microspheres obtained at drug/copolymer ratio of 1:16 (X_3_; +1) were characterized by the maximum particle size in every batch. High (+1) level of X_2_, and low (−1) level of X_1_ and X_3_ decreased particle size. When *n*BuOAc was used (X_1_; +1) at medium concentration (X_2_; 0), microspheres were formed with the maximum particle size (278–313 μm), because the increase in the CH_2_Cl_2_-*n*BuOAc viscosity resulted in merged droplets or in a reduction of the efficiency of disruption of the W_1_/O emulsion into droplets. The trends observed for the various batches were practically the same: particle size at constant X_2_ increased with increasing X_3_ level, while at constant X_3_ and increasing X_2_ levels, particle size increased up to 50% cosolvent content, and dropped at 75% content, due to the decreased viscosity of the emulsion droplets. The negative sign of the X_2_ effect (b_2_: −15.05) confirmed this incident.

### 3.7. Y_4_ Response: Investigation of Drug Encapsulation Efficiency (EE)

The value of EE is the result of a sensitive balance between two main key factors as opposite effects, the rate of CH_2_Cl_2_+cosolvent migration to the W_2_ phase and the duration of copolymer precipitation.

Addition of a polar cosolvent and therefore fast partitioning and extraction could decrease the interfacial tension between the organic and aqueous phases, and form a dense wall, which can prevent the confluence of the aqueous phases, and ensure a dense microparticle structure with high EE [34]. Rapid increase in the η viscosity led to a reduction in the drug partitioning into the W_2_ phase, and the more viscous W_1_/O emulsion was less fragmented; these effects resulted in drug retention [2]. On the other hand, the addition of a cosolvent could increase the microsphere porosity, leading to drug loss during the preparation and therefore a lower EE [34]. On the basis of preliminary studies [35] the used drug/copolymer ratios can ensure the molecular dispersion of drug in the copolymer matrix.

EE varied in the ranges of 10.5–53.3%, the factorial design indicated a good fit (R^2^ = 0.978). Cosolvent log P at all levels, cosolvent concentrations at medium or high (X_2_; 0 and +1) levels and low drug/copolymer ratio (X_3_; −1) yielded microspheres with the highest EE (33.4–53.3%). The appreciable effects of X_2_ (b_2_: 3.45) and X_3_ (b_2_: −10.9) on EE indicated main effects that differed in magnitude and mathematical sign.

The use of polar cosolvents ensures a driving force for CH_2_Cl_2_ and the W_1_ phase to enter the W_2_ phase. A high (+1) level of X_2_ and a low (−1) level of X_3_ led to the maximum of EE, which confirmed that the polar cosolvent can leave the W_1_/O emulsion faster, resulting in the fast solidification of the copolymer and in more drug in the W_1_ droplets. Moreover the droplets may have remained in the liquid form for a longer period of time if *n*BuOAc were used, leading to a greater drug leakage, which was reflected in the decreased EE values (11.4–38.4%); however the more viscous W_1_/O emulsion could be less likely fragmented, resulting in drug retention.

### 3.8. Y_5_ Response: Investigation of Cumulative Drug Release (Q_6_)

In this study, significant effect of cosolvent logP (cosolvent type) on drug release, and significant but less determinant the cosolvent and drug/copolymer ratios, was observed. Q_6_ varied in the ranges of 3.26–100%. The preliminary thermoanalytical and Raman spectroscopical preformulation studies showed stable character of the drug in the microspheres and revealed an absence of considerable drug-copolymer interaction, which could be responsible for the additional retaining effect of the drug, altering the drug release rate [36]. The release pattern was found to be complex, the goodness of fit for the kinetic models used ranked in the sequence of Hixson-Crowell < Baker-Lonsdale ≅ Higuchi < Nernst. The Nernst dissolution profile best followed the release profile of *S0A*-*S0C*; after a slow dissolution the release rate reached a plateau (R^2^ > 0.955). The absence of a burst effect could be due to the preferential location of drug inside the deep sections of the copolymer matrix. For batches without a burst effect, the Baker-Lonsdale and Higuchi models were found to provide best fit. Batches reaching a plateau after 2 h conformed to the Hixson-Crowell model (R^2^ > 0.95). The absence of an initial burst was observed for batch *S1*-*S9*; the rapid Me_2_CO diffusion could lead to a dense copolymer matrix, eliminating the burst release, and thus the rate of drug diffusion was attenuated (Q_6_: 3.2–48.6 h). In contrast, a high burst release was observed for batch *S19*-*S27* (*n*BuOAc) (Q_6_: 47.1–100.0 h). Pore diffusion, disruption or disintegration of the matrix, as expressed in the burst effect, became more predominant at high drug/copolymer ratio.

Due to the relatively low Q_6_ values, the CH_2_Cl_2_-Me_2_CO mixture could be useful when sustained release for a longer period is the required dissolution profile. At constant X_2_, an increase of X_3_ was found to improve the dissolution of drug appreciably. The release profiles of *S1*-*S5* proved linear, conforming the Higuchi equation (R^2^ > 0.973), and *S6*-*S9* followed the Hixson-Crowell release profile (R^2^ > 0.932). This confirmed dissolution rate limitation of drug release from microparticles and revealed to no dramatic changes in the structure of them meanwhile [37].

*S10*-*S12* (X_2_; −1) fitted the Baker-Lonsdale model (R^2^ > 0.941), describing release profiles from matrices with uniform drug distribution, while the release profile of *S14*-*S16* (X_2_; 0, +1) fitted the Nernst model (R^2^ > 0.962). *S17*-*S18* (X_2_; 0, +1) did not meet the requirements (max. 80% in 6 h).The *n*BuOAc has the highest boiling point and viscosity of the cosolvents used; since the rate of evaporation of the solvent depends on its boiling point, the influence of the slow evaporation combined with the higher viscosity was more evident for this batch, resulting in microspheres with a large specific surface area, low EE, a porous nature and hence a high release rate with initial burst. A possible reason for the high drug release could be the formation of large pores and deep channels, explained by the rapid extraction of *n*PrOH from the W_1_/O emulsion, which may act in this way as an effective pore-forming agent. Q_6_ was accompanied by a burst release effect, followed by the sustained release of 70–86% over 6 h. The release from *S20*-*S21* and *S23*-*S24* (X_3_: 0;+1) was fast (t_90_ < 0.70 h), the amounts released exceeded the aims, the surface defects could be responsible for the burst effect. *S19*, *S22* and *S25*-*S27* (X_3_; −1) satisfied the Nernst equation with a good fit (R^2^ > 0.977).

**Figure 4.**
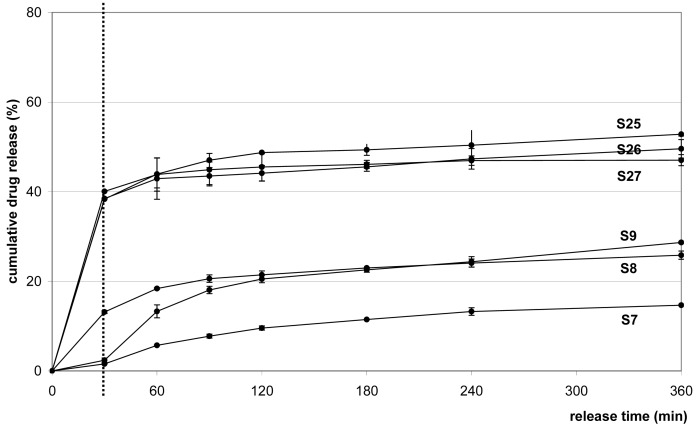
Effect of drug/copolymer ratio (factor X_3_) on rate of drug release (X_1_; X_2_; X_3_): *S7* (−1; +1; −1), *S8* (−1; +1; 0), *S9* (−1; +1; +1), *S25* (+1; +1; −1), *S26* (+1; +1; 0) and *S27* (+1; +1; +1).

X_1_ proved statistically significant in its linear (b_1_: 31.07), quadratic (b_11_: 10.58) and interaction (b_12_: −14.34) effects. The joint effects of X_1_ and X_2_ on the cumulative drug release rate are reflected by the following representative release profiles (b_12_ = −14.34), where *S1*, *S10* and *S19* (X_2_ = −1) and *S7*, *S16*, *S19* (X_2_ = +1), when X_3_ was kept constant (−1). In spite of their different release behavior, the production yields (62–74%), particle size (108–205 μm), and EE (33–53%) values of these batches were similar; thus, mainly the log P and concentration of the cosolvents appeared to determine the drug release. Figure 4 demonstrates the effects of X_3_ on the cumulative drug release for *S7*–*S9* and S25–S27 (X_2_ = +1; X_3_: −1 → +1). The similar release profiles indicated that the release rate could be modified only slightly by varying the drug/copolymer ratio (b_3_ = 5.71, R^2^ < 0.900).

### 3.9. Organic Solvent Residue

A relatively low amount of organic solvent residue could be achieved by increasing the temperature of spray drying, but the amount of the organic polar cosolvent residue depends even more on its affinity to the polymer. The concentration limit (ppm) and PDE (permitted daily exposure) of CH_2_Cl_2_ are 600 ppm and 6.0 mg day^−1^, respectively [38]. The maximum residual CH_2_Cl_2_ content in the microspheres prepared with 100% v/v CH_2_Cl_2_ (*S0A*–*S0C*) was 808.5 ppm (S.D.: 3.81%), because the duration of spray-drying process could be short to eliminate the residual CH_2_Cl_2_, it has been examined in our previous study [39].

Class 3 solvents, used as cosolvents in this work, have a concentration limit of 5000 ppm (PDE = 50 mg day^−1^) [38]. The maximum residual CH_2_Cl_2_ content was <500 ppm and the maximum concentrations of the cosolvent residues in the microspheres prepared at high (+1) X_2_ rate were 441.48 (Me_2_CO), 1796.39 (MeCOEt) and 954.0 ppm (*n*BuOAc) (S.D.: 1.77–6.12%), which met the requirements. These results confirmed that the amount of cosolvent residue did not depend on the boiling point or the extraction rate. The reason for the relatively high residual amounts of MeCOEt and *n*BuOAc was their higher lipophilicity, and thus the slower saturation of the W_2_ phase.

## 4. Conclusions

The individual and joint effects of independent variables {log P of cosolvent (X_1_); cosolvent concentration (X_2_); and drug/copolymer ratio (X_3_)} on the properties of spray-dried AMC-based microspheres were investigated with a factorial-based design. This work is based on the fact that the use of polar cosolvents in the multiple emulsion method can increase the risk of confluence of the aqueous phases, and thus the decreased stability of the multiple emulsion could cause appreciable alterations in the microsphere properties.

The factorial design allowed selection of the levels of the independent variables yielding an optimum microsphere product. Table 5 summarizes the optimization process between the required levels of the independent variables, furnishing a basis for predictions of further quantitative data. Low and medium (−1;0) levels of X_1_, X_2_ and X_3_, as independent variables, were used to obtain microspheres with a relatively high production yield (Y_2_: 45.1–71.5%), low particle size (Y_3_: 120.6– 205.4 μm) and high EE (Y_4_: 41.8–53.3%). For sustained and relatively low drug release, MeCOEt as cosolvent was appropriate at low and medium (−1 and 0) levels of X_2_ and X_3_, as was Me_2_CO at medium level (0) of X_2_ factor. The following results were obtained as concerns the independent variables:

### —Log P of cosolvent (X_1_)

The CH_2_Cl_2_-cosolvent composition was the key factor controlling the properties of the microspheres according to the demand of the formulator. Although the effects of the polar cosolvents used proved complicated, linear relationship was observed between the cosolvent properties and the measured responses, indicating a strong influence of the W_1_/O viscosity on the microsphere physicochemical parameters. Me_2_CO and MeCOEt were clearly the best cosolvents in this work, the examined dependent variables reaching the optimum. Me_2_CO and MeCOEt best increased the precipitation of the copolymer, ensured low W_1_/O viscosity and increased the hardening of copolymer, indicating the final sequence *n*BuOAc < Me_2_CO ≈ MeCOEt in accordance with the aims.

### —Concentration of cosolvent (X_2_)

A medium concentration of the less toxic and more polar cosolvents had a much higher positive effect than a high concentration. However, the cosolvents at a high concentration ratio were found to leave low residual impurities. Conversely, X_2_ at high (+1) level, in spite of the rapid preparation process, the less stable W_1_/O emulsion droplets could not retain the drug inside during preparation, and EE decreased due to the osmotic effect of the W_1_ phase.

### —The drug/copolymer ratio (X_3_)

The sequence 1:32 < 1:24 ≈ 1:16 for drug/copolymer ratio made this variable optimum, for optimization of the microsphere characteristics, X_3_ at medium and high level proved most effective. At this ratio, the W_1_ droplet size decreased the possibility of confluence of the aqueous phases, improving EE. Future studies appear important to increase the EE values.

## Figures and Tables

**Figure 3.**
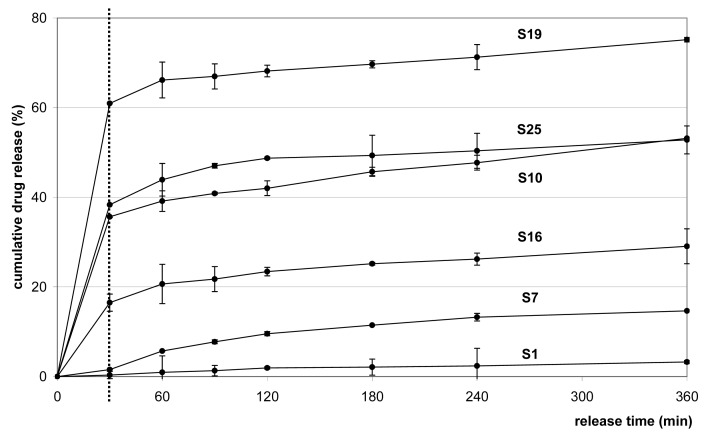
Effect of cosolvent log P (factor X_1_) and concentration (factor X_2_) on rate of drug release (X_1_; X_2_; X_3_): *S1* (−1; −1; −1), *S7* (−1; +1; −1), *S10* (0; −1; −1), *S16* (0; +1; −1), *S19* (+1; −1; −1) and *S25* (+1; +1; −1).

**Table 1.** Physicochemical properties of the organic solvents used.

**Used solvents**	**ICH Class**	**Log P**	**Boiling point (°C)**	**Visc.^a^ (mPas)**	**Sol. in water (g·100 mL^−1^)**	**Saturation^b^**
Me_2_CO	3	0.234	56.5	0.360	miscible	Mixing
MeCOEt	3	0.736	79.6	0.415	29.0	-
CH_2_Cl_2_	**2**	1.511	39.5	0.475	1.3	Rapid
*n*BuOAc	3	1.822	125.0	0.730	0.7	Rapid

a absolute viscosity data from preliminary measurements (relative density of water = 1.000);

b saturation at maximum cosolvent concentration (75% v/v) in the aqueous phase.

**Table 2.** Levels and values of the independent variables (non-randomized).

**Levels**	**Values**		
	**X_1_ (log P)**	**X_2_ (cosolvent conc.) (% v/v)**	**X_3_ (drug/copolymer ratio)**
**−1**	0.234 (Me_2_CO)	25	1:32
**0**	0.736 (MeCOEt)	50	1:24
**+1**	1.822 (*n*BuOAc)	75	1:16

**Table 3.** The microsphere batches investigated in factorial design.

**X_1_**									
(−1)	S1	S2	S3	S4	S5	S6	S7	S8	S9
(0)	S10	S11	S12	S13	S14	S15	S16	S17	S18
(+1)	S19	S20	S21	S22	S23	S24	S25	S26	S27

**X_2_**	−1			0			+1		

**X_3_**	−1	0	+1	−1	0	+1	−1	0	+1

The batch of *S0A*-*S0B*-*S0C* was prepared with CH_2_Cl_2_ alone with the levels of X_3_: −1/0/+1, respectively.

**Table 4.** Coefficients for the mathematical models. Linear (b_0_–b_3_), synergistic (b_12_–b_23_) and quadratic (b_11_–b_33_) effects of the independent variables on the dependent variables (Y_1_–Y_5_).

**Resp.**	**b_0_**	**b_1_**	**b_2_**	**b_3_**	**b_12_**	**b_13_**	**b_23_**	**b_11_**	**b_22_**	**b_33_**	**R^2^**
**Y_1_**	**12.49**	**4.68**	**−2.22**	**−1.25**	**0.30**	**−0.49**	**0.53**	**−1.74**	**−0.32**	−0.07	**0.9988**
**Y_2_**	**59.05**	**−10.38**	**1.86**	**−6.63**	**3.93**	−0.39	0.00	**5.35**	**−3.39**	−0.64	**0.9449**
**Y_3_**	**186.07**	**26.71**	**−15.05**	**20.17**	**−23.29**	−0.93	−1.69	**−7.88**	**27.31**	2.12	**0.9875**
**Y_4_**	**26.76**	**−1.82**	**3.45**	**−10.9**	−0.66	**2.72**	−1.21	**1.66**	−0.76	**−1.46**	**0.9784**
**Y_5_**	**51.64**	**31.07**	−0.41	5.71	**−14.34**	0.25	4.44	**10.58**	4.41	3.58	**0.9057**

*Y_1_*: W_1_/O viscosity (mPas); *Y_2_*: Production yield (%); *Y_3_*: Average particle size (μm); *Y_4_*: EE (%); *Y_5_*: Cumulative release in 6h (μg/mL)

**Table 5.** Optimization of levels of independent variables according to required effects.

	**X_2_** (−1)	**X_2_** (0)	**X_2_** (+1)	
**X_1_** (−1)	Y_1_-Y_4_	Y_1_-Y_4_	*Y_1_*-*Y_4_*	**X_3_** (−1)
	Y_1_-Y_4_	*Y_1_*-*Y_5_*	*Y_1_*-*Y_3_*; *Y_5_*	**X_3_** (0)
	Y_1_-Y_4_	*Y_1_*-*Y_5_*	*Y_1_*-*Y_3_*; *Y_5_*	**X_3_** (+1)
**X_1_** (0)	*Y_1_*-*Y_5_*	*Y_1_*-*Y_5_*	Y_1_-Y_4_	**X_3_** (−1)
	*Y_1_*-*Y_5_*	*Y_1_*-*Y_5_*	Y_1_-Y_3_	**X_3_** (0)
	*Y_1_*-*Y_5_*	*Y_1_*-*Y_5_*	Y_1_-Y_3_	**X_3_** (+1)
**X_1_** (+1)	Y_2_-Y_4_	Y_2_; Y_4_	*Y_1_*-*Y_5_*	**X_3_** (−1)
	Y_3_-Y_4_	Y_4_	Y_1_; Y_3_; Y_5_	**X_3_** (0)
	Y_3_-Y_4_	Y_4_	Y_1_; Y_3_; Y_5_	**X_3_** (+1)

Required effects, as Y_1_—low W_1_/O viscosity; Y_2_—high production yield; Y_3_—low particle size; Y_4_—high EE and Y_5_—Q_6_ 40–80% in 6 h, can be ensured by the compositions highlighted.

## References

[1] Godbee J., Scott E., Pattamunuch P., Chen S., Mathiowitz E. (2004). Role of solvent/non-solvent ratio on microsphere formation using the solvent removal method. J. Microencapsul..

[2] Sohier J., van Dijkhuizen-Radersma R., de Groot K., Bezemer J.M. (2003). Release of small water-soluble drugs from multiblock copolymer microspheres: a feasibility study. Eur. J. Pharm. Biopharm..

[3] Constantin M., Fundueanu G., Bortolotti F., Cortesi R., Ascenzi P., Menegatti E. (2004). Preparation and characterisation of poly(vinyl alcohol)/cyclodextrine microspheres as matrix for inclusion and separattion of drugs. Int. J. Pharm..

[4] Biju S.S., Saisivam S., Maria Gerald Rajan N.S., Mishra P.R. (2004). Dual coated erodible microcapsules for modified release of diclofenac sodium. Eur. J. Pharm. Biopharm..

[5] Lee E.S., Kwon M.J., Lee H., Na K., Kim J.J. (2006). *In vitro* study of lysozyme in poly(lactide-co-glycolide) microspheres with sucrose acetate isobutyrate. Eur. J. Pharm. Sci..

[6] Li Z., Li L., Zhang H., Li X., Luo F., Mei X. (2011). Development of interferon alpha-2b microspheres with constant release. Int. J. Pharm..

[7] Arica B., Kas H.S., Orman M.N., Hincal A.A. (2002). Biodegradable bromocryptine mesylate microspheres prepared by a solvent evaporation technique. I: Evaluation of formulation variables on microsphere characteristics for brain delivery. J. Microencapsul..

[8] Saravanan M., Anupama B. (2011). Development and evaluation of ethylcellulose floating microspheres loaded with ranitidine hydrochloride by novel solvent evaporation-matrix erosion method. Carbohydr. Polymer..

[9] Peltonen L., Koistinen P., Karjalainen M., Häkkinen A., Hirvonen J. (2002). The effect of cosolvents on the formulation of nanoparticles from low-molecular-weight poly(l)lactide. AAPS PharmSciTechn.

[10] Wang F.J., Wang C.H. (2002). Sustained release of etonidazole from spray dried microspheres prepared by non-halogenated solvents. J. Control. Release.

[11] Jose S., Prema M.T., Chacko A.J., Thomas A.C., Souto E.B. (2011). Colon specific chitosan microspheres for chronotherapy of chronic stable angina. Colloids Surf. B Biointerfaces.

[12] Li X., Zhang Y., Yan R., Jia W., Yuan M., Deng X., Huang Z. (2000). Influence of process parameters on the protein stability encapsulated in poly-DL-lactide-poly(ethylene glycol) microspheres. J. Control. Release.

[13] Jayant R.D., McShane M.J., Srivastava R. (2011). *In vitro* and *in vivo* evaluation of anti-inflammatory agents using nanoengineered alginate carriers: Towards localized implant inflammation suppression. Int. J. Pharm..

[14] Curcio M., Spizzirri U.G., Iemma F., Puoci F., Cirillo G., Parisi O.I., Picci N. (2010). Grafted thermo-responsive gelatine microspheres as delivery systems in triggered drug release. Eur. J. Pharm. Biopharm..

[15] Abdul S., Chandewar A.V., Jaiswal S.B. (2010). A flexible technology for modified-release drugs: Multiple-unit pellet system (MUPS). J. Control. Release.

[16] Zhang J., Wang Q., Wang A. (2010). *In situ* generation of sodium alginate/hydroxyapatite beads as drug-controlled release matrices. Acta Biomaterialia.

[17] Manconi M., Mura S., Sinico C., Fadda A.M., Vila A.O., Molina F. (2009). Development and characterization of liposomes containing glycols as carriers for diclofenac. Colloid. Surf. A.

[18] Kramar A., Turk S., Vrečer F. (2003). Statistical optimisation of diclofenac sustained release pellets coated with polymetacrylic films. Int. J. Pharm..

[19] González-Rodríguez M.L., Maestrelli F., Mura P., Rabasco A.M. (2003). *In vitro* release of sodium diclofenac from a central core matrix tablet aimed for colonic drug delivery. Eur. J. Pharm. Sci..

[20] Fujimori J., Yoshihashi Y., Yonemochi E., Terada K. (2005). Application of Eudragit RS to thermosensitive drug delivery systems. J. Control. Release.

[21] Trapani A., Laquintana V., Denora N., Lopedota A., Cutrignelli A., Franco M., Trapani G., Liso G. (2007). Eudragit RS 100 microparticles containing 2-hydroxypropyl-β-cyclodextrin and glutathione: Physicochemical characterization, drug release and transport studies. Eur. J. Pharm. Sci..

[22] Wu P.C., Huang Y.B., Chang J.S., Tsai M.J., Tsai Y.H. (2003). Design and evaluation of sustained release microspheres of potassium chloride prepared by Eudragit. Eur. J. Pharm. Sci..

[23] Año G., Esquisabel A., Pastor M., Talavera A., Cedré B., Fernández S., Sifontes S., Aranguren Y., Falero G., García L., Solís R.L., Pedraz J.L. (2011). A new oral vaccine candidate based on the microencapsulation by spray-drying of inactivated Vibrio cholerae. Vaccine.

[24] Kim B.K., Hwang S.J., Park J.B., Park H.J. (2002). Preparation and characterization of drug-loaded polymethacrylate microspheres by an emulsion solvent evaporation method. J. Microencapsul..

[25] Kumar V., Kang J., Yang T. (2001). Preparation and characterization of spray-dried oxidized cellulose microparticles. Pharmaceut. Dev. Technol..

[26] Huh Y., Cho H.-J., Yoon I.-S., Choi M.-K., Kim J.S., Oh E., Chung S.-J., Shim Ch.-K., Kim D.-D. (2010). Preparation and evaluation of spray-dried hyaluronic acid microspheres for intranasal delivery of fexofenadine hydrochloride. Eur. J. Pharm. Sci..

[27] Haswani D.K., Nettey H., Oettinger C., D'Souza M.J. (2006). Formulation, characterization and pharmacokinetic evaluation of gentamicin sulphate loaded albumin microspheres. J. Microencaps..

[28] Lee H.Y., Chan L.W., Dolzhenko A.V., Heng P.W.S. (2006). Influence of viscosity and uronic acid composition of alginates on the preparation of alginate films and microspheres produced by emulsification. J. Microencaps..

[29] Benita S. (1996). Microencapsulation: Methods and Industrial Applications.

[30] Ahuja N., Katare O.P., Singh B. (2007). Studies on dissolution enhancement and mathematical modeling of drug release of a poorly water-soluble drug using water-soluble carriers. Eur. J. Pharm. Sci..

[31] Lindenstruth K., Müller B.W. (2004). W/O/W multiple emulsions with diclofenac sodium. Eur. J. Pharm. Biopharm..

[32] Senuma Y., Lowe C., Zweifel Y., Hilborn J.G., Marison I. (2000). Alginate hydrogel microspheres and microspheres by spinning disk atomization. Biotechnol. Bioeng..

[33] Ruan G., Feng S.S., Li Q.T. (2002). Effects of material hydrophobicity on physical properties of polymeric microspheres formed by double emulsion process. J. Control. Release.

[34] Obeidat W.M., Price J.C. (2003). Viscosity of polymer solution phase and other factors controlling the dissolution of theophylline microspheres prepared by the emulsion solvent evaporation method. J. Microencapsul..

[35] Sipos P., Szűcs M., Szabó A., Erős I., Szabó-Révész P. (2008). An assessment of the interactions between diclofenac sodium and ammonio methacrylate copolymer using thermal analysis and Raman spectroscopy. J. Pharm. Biomed. Anal..

[36] Sipos P., Szabó A., Erős I., Szabó-Révész P. (2008). A DSC and Raman spectroscopy study of microspheres prepared with polar cosolvents by different techniques. J. Therm. Anal. Cal..

[37] Karasulu E., Karasulu H.Y., Ertan G., Kirilmaz L., Güneri T. (2003). Extended release lipophilic indomethacin microspheres: formulation factors and mathematical equations fitted drug release rates. Eur. J. Pharm. Sci..

[38] (2009). ICH Harmonised Tripartite Guideline. Impurities: Guideline for Residual Solvents, Q3C (R4).

[39] Sipos P., Csóka I., Srčič S., Pintye-Hódi K., Erős I. (2005). Influence of preparation conditions on the properties of Eudragit microspheres produced by a double emulsion method. Drug Dev. Res..

